# Making Muscle Elastic: The Structural Basis of Myomesin Stretching

**DOI:** 10.1371/journal.pbio.1001264

**Published:** 2012-02-14

**Authors:** Larissa Tskhovrebova, John Trinick

**Affiliations:** Astbury Centre for Structural Molecular Biology and Institute for Molecular and Cellular Biology, Leeds University, Leeds, United Kingdom

## Abstract

Skeletal and cardiac muscles are remarkable biological machines that support and move our bodies and power the rhythmic work of our lungs and hearts. As well as producing active contractile force, muscles are also passively elastic, which is essential to their performance. The origins of both active contractile and passive elastic forces can be traced to the individual proteins that make up the highly ordered structure of muscle. In this Primer, we describe the organization of sarcomeres—the structural units that produce contraction—and the nature of the proteins that make muscle elastic. In particular, we focus on an elastic protein called myomesin, whose novel modular architecture helps explain elasticity.

## Abstract 2

The muscle M-band protein myomesin comprises a 36-nm long filament made of repetitive immunoglobulin–helix modules that can stretch to 2.5-fold this length, demonstrating substantial molecular elasticity.

## Sarcomeres

**Figure 1:**
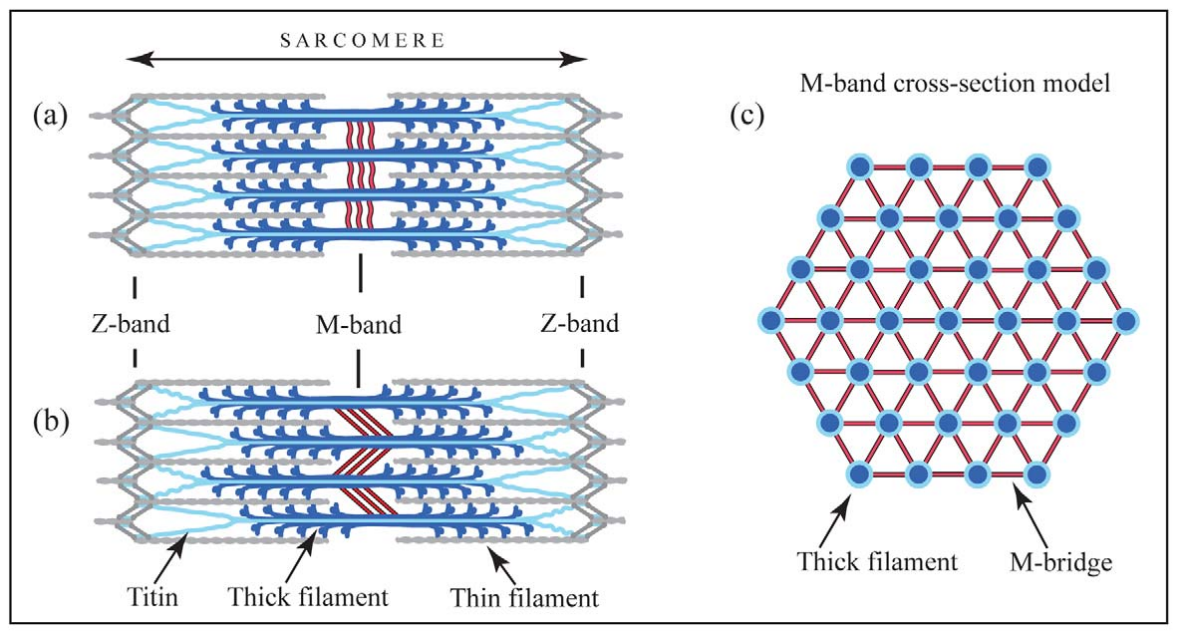
Schematics of sarcomere and M-band structure derived from electron microscopy of muscle [**1]**,[2****]. (a) Relaxed sarcomere. (b) After isometric contraction causing tension in the M-band and sliding between thick filaments; (c) M-band model showing M-bridges connecting thick filaments, as well as additional links connecting M-bridges.

Muscle fibres arise by fusion of differentiating cells to form long multinucleate cells. The nuclei migrate to the cell periphery to make room for parallel arrangement of myofibrils—hundreds or even thousands of myofibrils per cell —composed of a series of repeating contractile units, the sarcomeres, each ∼2 µm long (Figure 1).

Sarcomeres are truly remarkable in their almost crystalline order, combined with an ability to deform elastically. At the centre of each sarcomere is a side-by-side array of bipolar thick filaments formed mainly of ∼300 molecules of the motor protein myosin. The thick filament array is hexagonal in cross-section (Figure 1c) and is inter-linked at the middle by bridging proteins forming the M-band. There are three to five registers of M-bridges in each M-band, separated by ∼20-nm intervals [1]–[5], depending on the muscle type. Interposed with thick filaments from both sides are thin filament arrays made mainly of actin, which are cross-linked by the Z-band proteins (Figure 1a). Muscle contraction occurs when the thin filaments are pulled further into the thick filament array by repetitive interactions of myosin with actin, thereby shortening the sarcomere.

## Elastic Proteins

The elasticity of the sarcomere derives mainly from ancillary cytoskeletal proteins whose modular architecture and flexibility allow reversible extension by force. One such protein, titin (also known as connectin), the largest known polypeptide, is long enough for a single molecule to span between the M- and Z-bands, linking the thick and thin filament arrays longitudinally (Figure 1). The C-terminus of titin is located in the M-band, from where it runs along the thick filament to its end. Between the thick filament end and the Z-band, titin forms elastic connections. These connections help the thick filaments stay equidistant between Z-bands during active and passive changes in sarcomere length. Without this centering mechanism, force imbalances develop between opposite thick filament halves during contraction [6]. The mechanism of titin elasticity is hierarchical, using both the flexibility of the molecule and its ability to reversibly unfold. Shortening of the sarcomere is thought to force the molecule to coil up and, as a result, to develop a restoring force. Increases in sarcomere length first straighten titin and then extend its unstructured regions. Extension increases tension and the tendency to restore a compact shape.

Sarcomere contraction or passive extension also produces lateral forces leading to expansion or compression of the thick [7]–[10] and thin filament lattices [7],[11]–[15], requiring transverse elasticity in both the M- and Z-bands. X-ray diffraction of live resting muscle shows that thick filament spacing changes inversely proportional to the square root of sarcomere length [16]. Isometric contraction (when force is produced but no shortening allowed) expands the thick filament lattice with a radial force of ∼1 nN per thick filament, or ∼10 pN per myosin (in frog skeletal muscle) [15]. This shows that force expanding the thick filament array is high and will raise tension in the M-band and extend the M-bridges. During prolonged isometric contraction, thick filament centering by titin may fail, leading to loss of register between the filaments and widening and smearing of M-bands (Figure 1b) [6]. Sharp M-bands are restored during relaxation, demonstrating the elasticity of M-bridges.

M-bridges are made principally from isoforms of the myomesin family [17],[18] (see reference [19] for review), which are members of the immunoglobulin superfamily [20]. Myomesins are mainly products of the same gene [17],[18],[21]; however, one member of the family M-protein is encoded separately [22]. They are mostly located in the centre of the M-band, with only one found at its edge [23]. Isoforms in different muscles are also fibre-type dependent [24],[25].

Myomesins consist mainly of two domain types, immunoglobulin (Ig, C2-set) and fibronectin (type III, Fn3) [17],[18],[26],[27], both of which have ∼100 residues and are β-structures with seven or eight strands forming two sheets in a sandwich. There are seven Ig and five Fn3 domains in each molecule, arranged as Ig-Ig-Fn3-Fn3-Fn3-Fn3-Fn3-Ig-Ig-Ig-Ig-Ig. Their N-termini also have unique sequences that vary between isoforms. Myosin binds to both myomesin and M-protein near their N-termini: in myomesin, binding is to a unique sequence [28], whereas in M-protein it is through two Ig domains following the unique sequence [29]. Myomesin dimerizes through its C-terminal Ig domain, and this end-to-end dimer is suggested to form a single M-bridge [30]. Both myomesin and M-protein interact with muscle-type creatine kinase (MM-CK) through their central Fn3 domains, consistent with the presence of this enzyme in the M-band [31]. Other components probably enhance myomesin/M-protein binding to thick filaments, since interaction between myosin and M-protein alone does not form a stable complex [32].

## Myomesin Extensibility

Although several models for the arrangement of M-bridges have been suggested based on electron microscopy of muscle (Figure 1c) [1],[2],[5], mapped binding sites and antibody labelling patterns [25],[33], and self-interactions in myomesin [30],[34], the molecular architecture of the M-band remains unknown. However, in any arrangement of M-bridges, the mechanical properties of the individual components, such as myomesins, will determine the ability of the M-band to withstand deformation. Electron microscopy suggested flexibility in M-proteins [35],[36], probably arising from inter-domain mobility. This implies that passive coiling and uncoiling could provide low energy adaptation to weak forces. Recent single molecule mechanical experiments showed that embryonic myomesin isoforms have additional 100-residue-long unique sequences [37] likely to increase extensibility [38],[39]. However, as the adult isoforms lack similar unique sequences, the limits and mechanisms of myomesin extensibility in adult muscle remained unclear. The study by Pinotsis et al. in this issue of *PLoS Biology* [40] addressed this question. It concludes that α-helical interdomain linkers in myomesin are crucial: their rapid unfolding allows more than a doubling in length, while the β-structure domains and their interactions are preserved.

Pinotsis and colleagues explored myomesin extensibility in a combined structural and mechanical study of a five-domain C-terminal segment, My9–My10–My11–My12–My13 (My9–My13; in which the Ig-My13 domain is responsible for dimerization). The main technique used was X-ray crystallography, which typically attains the ∼0.3 nm resolution that allows the detail of the folded polypeptide structure to be correlated with sequence. Crystallization is difficult with large, flexible proteins, however, so Pinotsis et al. used a “divide-and-conquer” strategy to crystallize and examine three overlapping two- and three-domain fragments of My9–My13. Confirming the authors' expectations [34],[41], the resulting structures showed that all four linkers connecting Ig domains are α-helices of four or six turns. They termed the new modular arrangement IgH, emphasizing coupling between the β-structure Ig domains and α-helical linkers. The IgH unit (comprising one Ig domain and one helical linker) is relatively rigid and results in a roughly helical overall shape to the My9–My13 dimer, which is about 36 nm long.

The model constructed from the X-ray structures was independently validated by electron microscopy and small angle X-ray solution scattering (SAXS). Electron microscopy used the negative stain technique, which has a resolution of about 2 nm and is sufficiently detailed to allow individual protein shapes to be recognized. To assist in shape interpretation, maltose-binding protein (∼40 kDa) was fused to the N-terminus of My9–My13, which resulted in distinctive extra mass at the ends of dimer images. Shape definition was improved by averaging 2,000 dimer images, which revealed a shape similar to the one constructed from the crystal structures. *Ab initio* modelling of SAXS data also suggested a roughly helical dimer ∼36 nm long, in agreement with the crystallography and electron microscopy models, but also suggesting a degree of flexibility. Mutations in the linkers confirmed their importance in maintaining the overall shape of the molecule.

To explore the mechanics of myomesin, single molecule pulling was carried out on the My9–My13 dimer by using atomic force spectroscopy (AFM). For this, dimers were adsorbed onto a nickel–nitrilotriacetic acid (NTA) surface through poly-His tags at their N-termini. Elasticity was explored by using the cantilever probe of the AFM to absorb the free end of a dimer and pull on it. Force-extension curves revealed a saw-tooth pattern of peaks, each peak reflecting unfolding of a β-structure domain, which is similar to what is seen pulling titin. An interesting difference in the My9–My13 force-extension curves, however, was the plateaus that preceded the series of saw-tooth peaks. The lengths of the plateaus and the relatively low force at which they were observed were consistent with unfolding of the α-helical linkers between Ig domains. Repeated stretching and relaxation suggested that linker unfolding is rapid and close to equilibrium, and that this mechanism can more than double the length of My9–My13 without unfolding Ig domains.

The study by Pinotsis et al. is a good example of the divide-and-conquer approach of exploring structure and mechanics in complex protein structures in great detail. The new data enrich our knowledge of the mechanics of the modular proteins forming the muscle cytoskeleton. They suggest a simple mechanism of how the M-band absorbs tensile forces of transverse and longitudinal motions between thick filaments and keeps them in register. It has to be noted though that the myomesin fragment examined is less than half the molecule, and thus may not reflect its full mechanical potential. Schemes of myomesin arrangement suggest that M-band elasticity is likely to involve more than My9–My13. There is likely to be a hierarchy of events, starting with straightening molecules and followed by unfolding different regions depending on their relative mechanical strengths, as observed in titin-based sarcomere elasticity. An important prerequisite to a deeper understanding of sarcomere elasticity is also a higher resolution description of the sarcomere, sufficiently detailed to allow the shapes of the proteins to be recognized, from which we will be able to test the models inferred from component molecule studies.
